# Intercalated MOF nanocomposites: robust, fluorine-free and waterborne amphiphobic coatings†

**DOI:** 10.1039/d4en00762j

**Published:** 2025-01-29

**Authors:** Priya Mandal, Vikramjeet Singh, Jianhui Zhang, Manish K. Tiwari

**Affiliations:** a Nanoengineered Systems Laboratory, UCL Mechanical Engineering, University College London London WC1E 7JE U.K m.tiwari@ucl.ac.uk; b Wellcome/EPSRC Centre for Interventional and Surgical Sciences, University College London London W1W 7TS U.K; c Manufacturing Futures Lab, UCL Mechanical Engineering, University College London London E20 2AE U.K

## Abstract

Transparent non-wetting surfaces with mechanical robustness are critical for applications such as contamination prevention, (anti-)condensation, anti-icing, anti-biofouling, *etc.* The surface treatments in these applications often use hazardous per- and polyfluoroalkyl substances (PFAS), which are bio-persistent or have compromised durability due to weak polymer/particle interfacial interactions. Hence, developing new approaches to synthesise non-fluorinated liquid-repellent coatings with attributes such as scalable fabrication, transparency, and mechanical durability is important. Here, we present a water-based spray formulation to fabricate non-fluorinated amphiphobic (repellent to both water and low surface tension liquids) coatings by combining polyurethane and porous metal–organic frameworks (MOFs) followed by post-functionalisation with flexible alkyl silanes. Owing to intercalation of polyurethane chains into MOF pores, akin to robust bicontinuous structures in nature, these coatings show excellent impact robustness, resisting high-speed water jets (∼35 m s^−1^), and a very low ice adhesion strength of ≤30 kPa across multiple icing/de-icing cycles. These surfaces are also smooth and highly transparent, and exhibit excellent amphiphobicity towards a range of low surface tension liquids from water to alcohols and ketones. The multi-functionality, robustness and potential scalability of our approach make this formulation a good alternative to hazardous PFAS-based coatings or solid particle/polymer nanocomposites.

## Abstract 2

We report water-based transparent and non-fluorinated amphiphobic coatings using polyurethane and metal–organic frameworks (MOFs). These coatings are scalable, robust, and offer a safer alternative to PFAS-based treatments.

## Graphical abstract

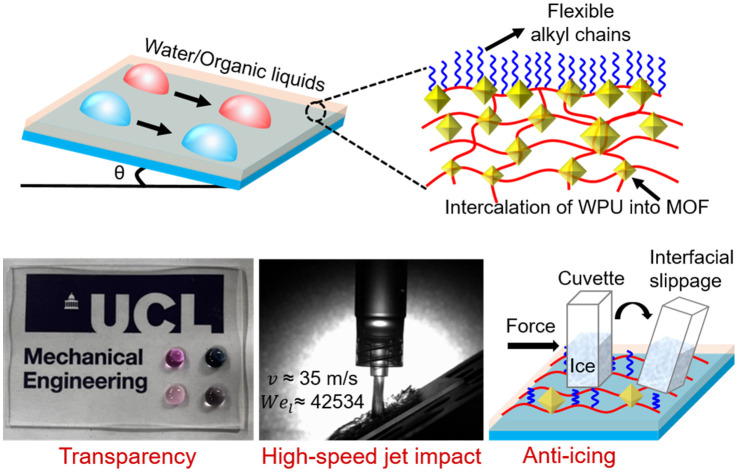

**Environmental significance**

The development of robust, fluorine-free, and waterborne liquid-repellent coatings marks a significant advancement in the pursuit of environmentally sustainable materials. Traditional coatings often rely on per- and polyfluoroalkyl substances (PFAS) to achieve liquid repellency, but these compounds are known for their persistence in the environment and harmful effects on ecosystems and human health. The move towards fluorine-free chemistries mitigates some of these issues by eliminating the source of PFAS contamination, thereby reducing the long-term environmental footprint. In addition, the conventional liquid-repellent coatings typically utilise organic solvents, which are sources of volatile organic compounds (VOCs). The emission of VOCs during application and drying contributes to air pollution and ozone formation, and poses direct health risks. Waterborne coatings, by contrast, significantly reduce VOC emissions, aligning with global efforts to protect air quality and human health. These advancements align with the UK's environmental policies and goals, including the push for net-zero emissions by 2050 and compliance with the European Union's REACH (Registration, Evaluation, Authorisation, and Restriction of Chemicals) regulation. By adopting PFAS-free waterborne coatings, we can lead in the transition towards safer and more sustainable chemical practices.

## 1. Introduction

The impact of global warming and climate change can be widely observed in the modern world.^1,2^ Both natural (*e.g.* fossil fuels, biomass, metals, *etc.*) and synthetic (*e.g.* chemicals, polymers, medicines, *etc.*) materials have strong and complicated interlinkage to the climate change.^3–6^ Innovation in materials and their processing such as the use of safe chemicals, efficient manufacturing processes, production of robust components, *etc.* have also attracted great attention due to the direct impact on some of the United Nations' sustainable development goals (SDGs): 6, 7, 9, 11, 12, 13 and 14.^7,8^

Similar to any other technical advances, fabrication of liquid-repellent materials (including coatings) has attracted the interest of the scientific community, targeting a wide variety of domestic and industrial applications in self-cleaning windows,^9^ contamination prevention,^10^ corrosion resistance,^11,12^ anti-biofouling,^13,14^ anti-icing,^15^*etc.* However, achieving repellence, especially towards low surface tension liquids *e.g.* oils or organic solvents generally requires perfluorinated compounds (PFCs) which are biopersistent (non-degradable in the environment) due to very strong carbon–fluorine bonds.^16^ In fact, there is widespread concern regarding an even broader class of chemicals, designated as per- and polyfluoroalkyl substances (PFAS) of which PFCs are a part of. Efforts have been made to produce amphiphobic surfaces/coatings using other strategies to avoid the use of hazardous PFAS in the wake of their adverse effect on the environment and human health, recently.^17,18^ For example, re-entrant surface structures reported by Wang *et al.* based on monodispersed large-sized silica particles showing superamphiphobic characteristics but lacking mechanical durability and robustness is a major challenge for its practical applications.^19^ In recent studies, Lai and co-workers have demonstrated different strategies, such as the use of interpenetrating polymer networks^20^ and dual cross-linked networks^21^ for robust fluorine-free liquid-repellent coatings. While these approaches achieve excellent mechanical durability and liquid repellency, they primarily rely on complex polymer architectures.

In a recent study, we reported transparent and robust amphiphobic surfaces exploiting nanohierarchical (a hierarchy of all-nanoscale roughness) surface-grown MOF films through layer-by-layer (L/L) growth.^22^ The characteristics of MOFs, including their large specific surface area and ultrahigh porosity, as well as their potential for chemical and structural modifications,^23,24^ make them excellent candidates for such applications. However, the L/L approach faces challenges in terms of scalability and therefore, a method that allow practical processability of bulk reticulated structures on surfaces is imperative. Additionally, achieving mechanically robust amphiphobic coatings using non-fluorinated materials, waterborne formulation, and scalable production methods is highly desirable but remains a major challenge.

The alternative nanocomposite-based coatings overcome the scalability issue; however, ensuring precise control of interfacial interactions between the polymer and nanoparticles is imperative, as it stands as a major source of (mechanochemical) weakness of the resulting nanocomposite. There are a number of natural examples from the dactyl club of mantis shrimps^25^ where a bicontinuous composite structure shows remarkable improvement in mechanical robustness and impact resistance characteristics. Drawing from this, we rely on a simple strategy in this paper to develop a nanocomposite where polymer chains are able to intercalate into the MOF pores. A simple, off-the-shelf waterborne polyurethane (WPU) and MOF nanoparticles are dispersed together followed by spraying to create nanocomposite coatings. This is followed by exploiting the hydroxyl groups of MOFs for post silanisation with flexible alkyl chains. Beyond simplicity and potential scalability of application, the resulting coating showed a number of striking properties. The nanohierarchical roughness enabled optical transparency as well as amphiphobicity, with excellent slipperiness to low surface tension liquids. The combination of these unique features makes this coating suitable for self-cleaning transparent surfaces (*e.g.*, solar panels and screens), anti-icing aerospace components, and durable automotive parts. Although MOF materials have higher preparation costs than traditional nanofillers, advancements in scalable synthesis methods, such as mechanochemical and continuous flow processes,^26,27^ along with the low MOF content (∼20 wt%) in the coating, enhance their cost-effectiveness and viability for large-scale applications. These coatings are clear at thicknesses up to tens of micrometres, can be applied readily onto a diverse range of substrates (such as smooth/textured, rigid/flexible, transparent/opaque, *etc.*) and are able to sustain extensive surface damage. The polymer intercalation and robustness enable the coatings to retain their amphiphobic characteristics even after being subjected to high-speed water jet impacts (*v* ∼ 35 m s^−1^) and repetitive icing/deicing cycles. The polymer intercalation is established through spectroscopic analyses, which demonstrate the superior performance of the MOF nanocomposite compared to silica nanoparticle-based composites. The coatings show thermal stability up to 200 °C (which is dictated by organic components) and stability to chemical exposure and repeated tape peel tests.

## 2. Experimental details

### 2.1. Materials

The water-based polyurethane (BAYHYDROL® UH 240) was provided by Covestro AG (Germany). Microscopic glass slides (75 mm × 25 mm) were purchased from Thorlabs. All the chemicals including zirconium chloride octahydrate (ZrOCl_2_·8H_2_O), terephthalic acid (TPA), dimethylformamide (DMF), acetone, ethanol, isopropanol, 1-butanol, glycol, glycerol, 1,2-butanediol, cyclohexanol, *n*-hexane, diiodomethane, and trichlorooctadecyl silane (OTS) were purchased from Sigma Aldrich. All the chemicals were used as received without further purification.

### 2.2. Instrumentation

The morphologies of all the samples (MOF powder and coated samples) were characterised using a scanning electron microscope (SEM, Carl Zeiss EVO25) and an atomic force microscope (AFM, Bruker ICON SPM). For SEM, the samples were coated with a thin gold film to avoid charging and observed at an accelerating voltage of 20 kV. To confirm the chemical composition, FTIR spectra were recorded on a PerkinElmer Spectrum Two™ spectrophotometer equipped with an iD5-ATR accessory, in a range of 4000 to 500 cm^−1^ at a resolution of 4 cm^−1^. The PXRD pattern (Stoe STADI-P) of the MOF was collected with CuKα radiation (*λ* = 1.542 Å) in the range of 5–40° with a step size of 5° min^−1^. A Raman spectrometer (Renishaw), equipped with a 532 nm argon-ion laser source with a power of 2.5 mW, was used to confirm the chemical structure of the MOF. The rheological properties of WPU and the composite coating dispersion were measured using a Discovery Hybrid rotational rheometer (DHR3, TA instrument). The viscosities were measured at different shear rates (from 0.1 to 1000 s^−1^) under ambient conditions (25 °C). Mechanical properties of WPU and intercalated WPU–MOF nanocomposite films were studied by a tensile test using an Instron 5659 testing machine provided with a 500 N load cell. Tensile strength, Young's modulus, elongation at break, and toughness were calculated from stress–strain curves. The UV-vis transmission spectra were recorded on an Orion™ AquaMate UV-vis spectrophotometer in the wavelength range of 300–800 nm.

### 2.3. Solid-state NMR

Solid-state NMR experiments were conducted on a Bruker Avance III HD spectrometer using a Bruker double-resonance 4 mm magic-angle spinning (MAS) probe. Quantitative ^13^C NMR spectra were acquired using multiCP at a spinning rate of 14 kHz, with a Hahn spin echo sequence generated by a 180° pulse and EXORCYCLE phase cycling. To ensure quantitative results, all multiCP samples were packed in HRMAS rotors. The *T*_1*ρ*_ spectra were obtained using cross-polarisation total sideband suppression (CPTOSS). The recycle delay and contact time were optimised for each sample, determined to be 4 s and 4 ms, respectively. All *T*_1*ρ*_ measurements were performed at a sample spin rate of 7 kHz, with a 40 kHz spin lock, and at a calibrated sample temperature of 21 °C.

^1^H direct excitation spectra were acquired with a 4 s recycle delay, calibrated for each sample, at a spinning rate of 10 kHz. Spectral referencing for ^13^C and ^1^H was done with respect to an external sample of tetramethylsilane, using the line from adamantane set to 38.5 ppm and 1.9 ppm, respectively.

### 2.4. MOF synthesis

MOF (UiO-66) were synthesised using an environment-friendly solvothermal process.^28^ In brief, 322.25 mg (25 mM) ZrOCl_2_·8H_2_O and 166.13 mg (25 mM) TPA were mixed in 40 mL DMF at room temperature and stirred for 5 minutes to achieve a clear solution. Then, the solution was sealed and placed in a pre-heated oven at 120 °C for 12 h. The crystallisation was carried out under static conditions. After cooling down to room temperature, the resulted mixture was centrifuged at 6500 rpm for 10 min and washed with DMF and acetone thrice. Then, the powder was dried in a vacuum oven at 80 °C overnight.

### 2.5. Spray coating and post-functionalisation

A measured amount of MOF nanoparticles (50–500 mg) were added to 1.0 g WPU and magnetically stirred for 20 min at 500 rpm to obtain a homogeneous dispersion. 20 mL water was added to the mixture and stirred for another 10 min to dilute the suspension. This diluted homogeneous mixture was stored in sealed glass bottles at room temperature for further use.

Prior to spraying, glass slides were cleaned ultrasonically using isopropanol and DI water for 15 minutes each, followed by drying with a N_2_ flow. The diluted homogeneous mixture was sprayed on glass slides at 2.5 bar pressure from a working distance of 15 cm using an air gun (Iwata Eclipse, ECL2000, nozzle diameter = 0.5 mm). During spraying, the glass slides were fixed vertically on a hot plate at 90 °C to escalate the water evaporation and then the coating was cured at 90 °C for another one hour. Further, the surface chemistry was modified by spraying 1% of OTS solution (isopropanol : water = 7 : 3) followed by curing at 120 °C for 2 h. The same process was followed to coat other substrates such as metals and plastics.

### 2.6. Contact angle measurement

The dynamic contact angles were measured using a custom-made goniometer setup.^29^ The setup consisted of an adjustable stage, syringe pump (World Precision Instruments, Aladdin single-syringe infusion pump), retort stand, a light source (Thorlabs, OSL2), and a zoom lens (Thorlabs, MVL7000) fitted to a CMOS camera. The videos of the droplets were analysed using ImageJ software to calculate advancing (*θ*_Adv_) and receding (*θ*_Rec_) contact angles. The reported values are the average of at least five measurements at different locations on a surface.

### 2.7. High-speed jet impact and tape peel test

High-speed jet impact experiments were performed to access the impalement resistance of the coating using a setup described elsewhere.^29^ A high-pressure nitrogen gas cylinder connected to an electronic pressure valve was used to force water through a nozzle (a needle/syringe assembly). Different nozzles with nominal diameters of 2.5 mm and 0.5 mm were used and the jet speed was controlled by tuning the gas pressure. For the 2.5 mm nozzle, the maximum jet speed achieved in our experiment was 35 m s^−1^ with the corresponding Weber number, We_l_ = 42 534. The coating showed no signs of liquid impalement even after repeated jet impact on the same spot. The lack of liquid impalement was confirmed by droplet mobility test.

To evaluate the mechanical durability of the coatings, pressure-sensitive and strong adhesive tape (3 M VHBTM tape 5952 with an adhesive peel strength of 3900 N m^−1^) was applied to the coatings and pressed evenly using a 2 kg roller to ensure adhesion to the coatings and peeled off after 60 s. This is considered as one cycle and the contact angle measurements were performed after each cycle to check the durability of the coating.

### 2.8. Thermal stability and ice adhesion measurement

Thermal stability of WPU–MOF coatings was evaluated by heating the samples from 40–200 °C for 1 h on a temperature-controlled hotplate and the droplet mobility (sliding angle) was tested after cooling down to ambient temperature. Change in contact angle hysteresis (Δ*θ*) was measured to quantify the droplet mobility after thermal treatment.

The ice adhesion strength of the coating was measured using a custom designed bench-top icing chamber whose details are described in our previous report.^22^ The samples were fixed to the base plate, and plastic cuvettes with a base area of 1 cm × 1 cm were placed on them. The entire chamber was cooled down to −15 °C using a refrigeration unit (FP50-HL refrigerated/heating circulator, Julabo). Then, water was poured into the cuvettes and the temperature was maintained for 2 h to ensure complete freezing of water. An extension rod connected to a force gauge (M4-50, MARK-10) was used to apply a shear force to the cuvettes and the peak force required to remove the ice was recorded. The ice adhesion strength was calculated by normalising the maximum force (*F*_m_) required to remove the ice with the area of cuvettes (*τ*_ice_ = *F*_m_/*A*, where *A* is the contact area between cuvettes and the coating surface). At least three parallel samples were measured to obtain an average adhesion value.

## 3. Results and discussion

### 3.1. Design principle and morphology

**Fig. 1:**
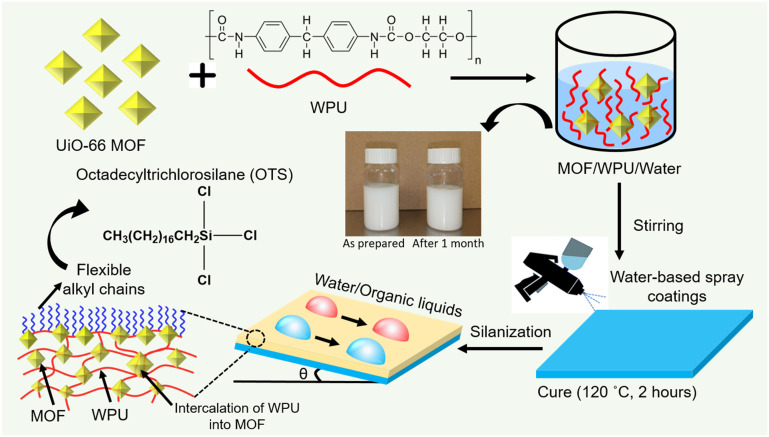
Design principle. Schematic illustration of sequential spray deposition of waterborne amphiphobic coatings.

The formulation and application strategy of the coating is shown in Fig. 1. Owing to their excellent thermal and mechanical stability, zirconium-based MOFs (UiO-66) were utilised as nanoparticles loaded in the WPU matrix.^30^ The crystallinity and chemical composition of the as-synthesised MOF nanoparticles were confirmed by PXRD and Raman spectra (Fig. S1 and S2,† respectively). The PXRD spectra of the MOF presents three characteristic diffraction peaks (2*θ*) at 7.3°, 11.96°, and 25.5°, which are in good agreement with the literature.^28^ Commercially available WPU (Bayhdrol® UH 240, Covestro) was selected as the polymer matrix over alternatives like polyolefins due to its superior flexibility, impact resistance, inherent transparency, and compatibility with MOF for effective intercalation.^31^ The nanoparticle concentration was adjusted to 20% by weight to achieve the best transparency, liquid-repellence, and mechanical robustness through the intercalation strategy conceived. Relatively higher concentration of nanoparticles, to ∼40%, resulted in an increase in Δ*θ* from ∼9° to ∼24° (Fig. S3†). The optimal dispersion is stable (stored over a month at room temperature) as confirmed by rheological measurements. The viscosity measurements indicate that the solution exhibited minimal variation over the storage period (Fig. S4†), with only a minor increase at higher shear rates. This observed increase is attributed to shear-thickening behavior, a phenomenon typically seen in concentrated polymer particle suspensions where the alignment of particles under shear leads to transient network formation, resulting in elevated resistance to flow.^32^ These results indicate that the precursor solution maintained its stability without significant changes in its rheological characteristics. The dispersion can be sprayed on different substrates, such as glass, metals, and polymers (Fig. S5†), which demonstrate the substrate independence of the coatings.

**Fig. 2:**
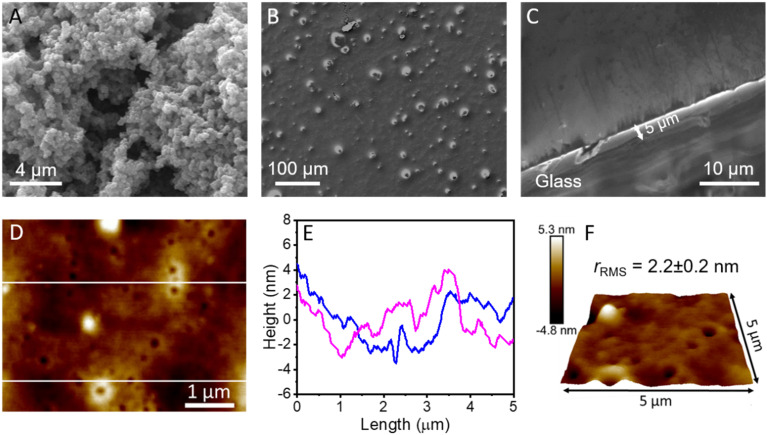
Surface morphology. SEM images of the (A) MOF nanoparticles and (B) WPU–MOF coating on glass. (C) Cross-sectional SEM image of the WPU–MOF coating on glass. (D) 2D topographical feature of the coating recorded using AFM. (E) Height profile recorded along the two white lines in D. (F) 3D AFM topography confirming the nanoscale roughness.

The surface morphology of the MOF and the coating was characterised using scanning electron microscopy (SEM). The as-synthesised MOF exhibited octahedral crystal structures with an average size of ∼100–200 nm (Fig. 2A). Fig. 2B confirms the uniformity of the spraying process and revealed the presence of well-dispersed MOF nanoparticles within the coating. The thickness of the sprayed composite was adjusted through a number of spray passes (∼20) to achieve a trade-off between the transparency, mechanical robustness, and liquid repellence (Fig. S6†). The cross-section of the optimised coating on glass was imaged under SEM and the thickness of the coating was measured to be ∼5 μm (Fig. 2C).The coating appeared homogeneous and smooth with very low local surface roughness scanned under an atomic force microscope (AFM). Fig. 2D–F show the 2D morphology, corresponding height profile, and 3D topography of the coating obtained from AFM. The root-mean square roughness (*r*_RMS_) was measured to be ∼2.2 nm (scan area = 5 × 5 μm^2^). The low roughness and flexibility from grafted silanes might have helped in promoting the liquid-repellence and transparency of the coating.

### 3.2. Transparency, chemical composition, and mechanical properties

**Fig. 3:**
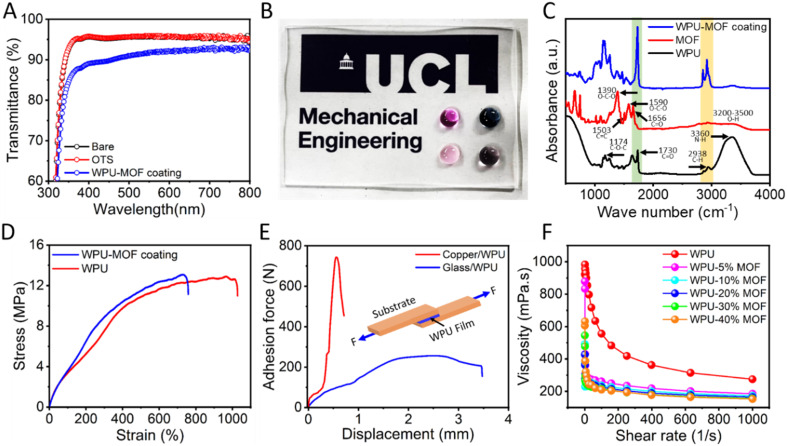
Transparency and mechanical properties. (A) The UV-vis transmission spectra of different samples: glass as a control (bare), trichlorooctadecyl silane coated glass (OTS), and WPU–MOF coating on glass. (B) Optical image showing droplets of water and other low surface tension liquids on the transparent WPU–MOF coating. (C) FTIR spectra of WPU, MOF, and the intercalated WPU–MOF nanocomposite. (D) Stress–strain curves of WPU and the intercalated WPU–MOF nanocomposite (standard dumbbell shape specimen, ASTM D412, obtained at a constant deformation speed of 1 mm min^−1^). (E) Adhesion force–displacement curves for glass and metal (copper) lap joints. The inset is the schematic of the sandwich specimen for the lap shear test. (F) Shear viscosity of WPU and the intercalated WPU–MOF nanocomposite dispersion as a function of nanoparticle concentrations.

The optical transparency of the coating was measured using UV-vis spectroscopy in transmittance mode and the obtained spectra are shown in Fig. 3A. The WPU–MOF coating exhibited >87% transmittance in the 400–800 nm spectral range with maximum 91% transmission. The transparency was also reflected by the easy visibility/readability of the characters underneath the coated surface, and the amphiphobicity is demonstrated by placing droplets of different low surface tension liquids such as water, ethylene glycol, glycerol, and butanol (Fig. 3B). The submicron chemically homogeneous and highly porous nanoparticles (MOFs) avoided the light scattering – resulting in enhanced transparency. The WPU, MOF, and intercalated WPU–MOF nanocomposite (cured) were examined using FTIR spectroscopy to confirm their chemical structure and identify the possible non-covalent interactions between the two components in the nanocomposite (Fig. 3C). The characteristic peaks at 3360 cm^−1^ (N–H stretching), 2938 cm^−1^ (C–H stretching), 1730 cm^−1^ (C=O stretching), and 1174 cm^−1^ (O–C–O) confirmed the chemical structure of WPU.^33^ The spectral band at 1656 cm^−1^ was attributed to stretching vibrations of C=O in the carboxylic acid of the MOF linker.^34,35^ In the case of the intercalated WPU–MOF nanocomposite, the C–H stretching of WPU centred at 2938 cm^−1^ and C=O vibrations centred at 1730 cm^−1^ were slightly shifted to 2916 cm^−1^ and 1726 cm^−1^, respectively, with a significant increment in the peak intensity. These changes might have occurred due to the physical interactions of WPU and the MOF caused by van der Waals, London dispersion, and electrostatic forces.^35^

The mechanical properties of the intercalated WPU–MOF nanocomposite, including stiffness and substrate adhesion, were measured to confirm the robustness of the coating. The tensile strength, Young's modulus, elongation at break, and toughness obtained for WPU and the intercalated WPU–MOF nanocomposite (Fig. 3D) are summarised in Table 1. The addition of the MOF into the WPU matrix has resulted in a slight increase in the tensile strength and Young's modulus of the film. However, the incorporation of the MOF led to smaller elongation at break, reduced from 1028% to 758% and toughness was also reduced by 22.5%. This was expected as the MOF can act as a reinforcement to the polymer matrix which makes the nanocomposite film more rigid than that obtained from only WPU. The high adhesion of WPU was tested on two different substrates (glass and copper), as shown in Fig. 3E. The coating shows an interfacial bonding strength of 1.9 MPa and 0.5 MPa with the copper and glass substrates, respectively. The obtained excellent adhesion could be due to the hydrogen bonding from N–H and C=O functional groups and strong non-covalent interactions (*e.g.* van der Waals forces) from the benzene ring of WPU to the hydroxyl groups on the substrates.^36^ In the presence of the MOF in WPU, the interfacial strength of the coating decreased to 1.6 MPa with copper (Fig. S7†). The slight reduction in adhesion strength is likely due to the disruption of the uniform interaction between WPU and the substrate by the MOF nanoparticles. However, this does not significantly affect the coating's overall performance, as the nanocomposite retains sufficient adhesion for practical applications. The rheological properties of nanocomposites are known to be strongly dependent on shape, size, orientation, and dispersion of the nanoparticles.^37^Fig. 3F shows the shear rate dependent viscosity of the intercalated WPU–MOF nanocomposite as a function of nanoparticle concentration. At a shear rate of 100 s^−1^, the viscosity of WPU was 556 mPa s which gradually reduced to 210 mPa s with the increase in the nanoparticle concentration from 5 to 20 wt%, respectively. The addition of MOF nanoparticles led to shear thinning and no significant change in the viscosity was observed beyond 20 wt% nanoparticle concentration *i.e.* optimised nanoparticle concentration. At a high shear rate of 1000 s^−1^, the viscosities remain similar for each case irrespective to the nanoparticle concentrations. The interfacial layer (IL) of WPU plays a major role in determining the rheological behaviour of the nanocomposite.^38,39^ The IL, defined as the fraction of polymer chains in direct contact with the particle surface,^40^ can impact the mobility and entanglement of these chains with the bulk polymer matrix. Upon intercalation, a part of the polymer chain is likely to remain outside to maintain mobility, ensuring that the viscosity remain unaffected.

**Table 1:** Tensile properties of WPU and the intercalated WPU–MOF nanocomposite film

Sample	Tensile strength (MPa)	Young's modulus (MPa)	Elongation at break (%)	Toughness (MJ m^−3^)
WPU	12.6	6.3	1028	121.6
WPU–MOF	13.2	7.8	758	94.3

### 3.3. Surface wettability

**Fig. 4:**
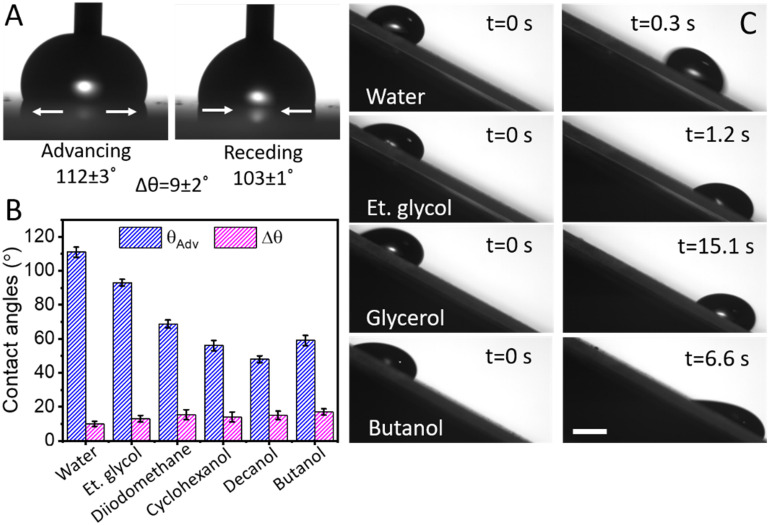
Surface wettability. (A) Advancing (*θ*_Adv_) and receding (*θ*_Rec_) contact angles of a water droplet on the intercalated WPU–MOF coating. (B) Bar diagram of *θ*_Adv_ and contact angle hysteresis (Δ*θ*) of water and different organic solvents with a wide range of surface tensions on the WPU–MOF coating; water (72.8 mN m^−1^), ethylene glycol (47.3 mN m^−1^), diiodomethane (50.8 mN m^−1^), cyclohexanol (32.9 mN m^−1^), decanol (28.5 mN m^−1^), and butanol (25.0 mN m^−1^). (C) Image sequence showing the amphiphobicity of the WPU–MOF coating through sliding of low surface tension liquid droplets (10 μL) at a 30° tilt angle. The low surface tension liquids are arranged with increasing viscosity (from top to bottom), which affect their sliding speed. The scale bar is 1 mm.

Irrespective of the substrate, the WPU–MOF coating appeared to be smooth with excellent repellence to a wide range of probing liquids. The dynamic wettability of the coated surfaces was assessed by measuring the advancing (*θ*_Adv_) and receding (*θ*_Rec_) contact angles, and contact angle hysteresis (Δ*θ*) of water droplets. As shown in Fig. 4A, the coating is hydrophobic with *θ*_Adv_ and Δ*θ* measured as ∼112 ± 3° and ∼9 ± 2°, respectively. Droplets of water and other low surface tension liquids such as ethylene glycol, glycerol, and butanol were observed to slide off at a <30° tilt angle (ESI† Video S1), demonstrating excellent amphiphobicity of the WPU–MOF coatings. The *θ*_Adv_ of the polar and non-polar liquids decreased with the reduction of surface tension (Fig. 4B). Δ*θ* values of low surface tension liquids were recorded to be slightly higher (∼15 ± 2°) when compared to water (∼9 ± 2°). The viscosity and surface energy of these liquids may explain this increase. Higher viscosity can slow down the movement of the contact line whereas lower surface energy of liquid enhances adhesion to the solid surface which results in higher Δ*θ*. The sliding behaviour of these liquids is shown in Fig. 4C and ESI† Video S1. To understand the nanohierarchical effect of MOF, only WPU coating (without MOF) after silanisation was also tested. Interestingly, WPU coating can only repel water but not the low surface tension liquids as shown in ESI† Video S2. In addition to nanohierarchical roughness, the controlled functionalisation of silane on the repeated crystalline units of embedded MOF contributes to amphiphobicity. This underscores the importance of nanohierarchical MOF (Fig. S8†) and the flexibility of the long silane chains which reduced the solid–liquid contact area and facilitated the easy sliding of liquid droplets.^22^

### 3.4. Mechanical robustness

**Fig. 5:**
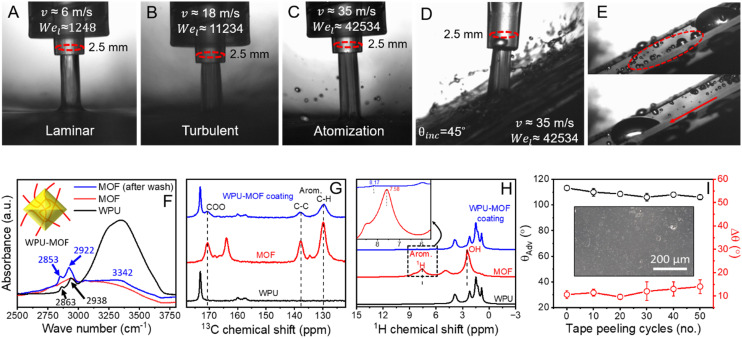
Mechanical robustness. (A–C) Snapshots of a 2.5 mm water jet impacting the WPU–MOF coating at ∼6 m s^−1^, ∼18 m s^−1^, and ∼35 m s^−1^, respectively. The jets are indicated as laminar, transitional, and turbulent depending on standard jet atomisation thresholds. (D) A turbulent water jet (∼35 m s^−1^) impacting the WPU–MOF coating inclined at 45°. (E) The coating showed no signs of impalement as tested with rolling droplets immediately after several jet impacts. (F) FTIR spectra of the WPU, MOF (as-synthesised), and WPU intercalated MOF. Inset: schematic representation of intercalation of WPU in the MOF. (G) ^13^C and (H) ^1^H NMR spectra of the WPU, MOF, and WPUMOF nanocomposite. Spinning frequency: 14 kHz. Inset of Fig. 5H: zoomed view of aromatic protons in the MOF and WPU–MOF coating. (I) The effect of tape peel cycles. Dynamic wetting behaviour remains unaffected even after 50 repeated cycles. Inset: SEM image of the coating after repeated tape peeling cycles.

Mechanical robustness is one of the major challenges in commercial applications. The substrate adhesion and inherent mechanical stability (cohesion) of coating components play a significant role in overall robustness.^41^ Therefore, in addition to nanohierarchical characteristics, zirconium-based MOF, with a high sheer modulus of 13.7 GPa, were chosen.^42^ Two different types of tests, high-speed jet impact and standard tape-peel were performed to assess the mechanical durability of the WPU–MOF coating. High-speed water jets were generated by pneumatic forcing of water through a nozzle to overcome the limitation of terminal velocity of gravity-accelerated drops (Fig. S9†).^29,43^ The coating was impacted with continuous water jets of different velocities and the events were captured by a high-speed camera shown in Fig. 5A–D and ESI† Video S3. The liquid Weber number (We_l_ = *ρv*^2^*d*/*γ*_LG_) was calculated to quantify the severity of the impact. With a 2.5 mm nozzle, the maximum speed achieved in our setup is ∼35 m s^−1^ with the corresponding We_l_ = ∼42 534. The jet forms a stagnation point at the point of impact and follows an axisymmetric stagnation flow trajectory. We also tested the ability of the WPU–MOF coating to withstand the repeated jet impacts at an average speed of 21 m s^−1^. After subjecting to repeated jet impacts, the coatings showed no damage or impalement by the liquid (Fig. S10†). Post-impact measurements did not show significant changes in the wettability either. The liquid impalement resistance was confirmed by placing a water droplet at the impacted area followed by normal sliding (Fig. 5E and ESI† Video S4). The impact resistance of the coating can be explained by comparing the capillary pressure (*P*_c_) of the MOF pores with the water hammer pressure (*P*_h_) generated in the jet impact. The capillary pressure from pores needs to resist the compressive water hammer pressure in order to avoid the liquid impalement.^44^ The capillary pressure can be calculated using eqn (1):

1 *P*_c_ = 4*γ*_LG_ cos *θ*_Adv_/*D*

Here, *D* is the average capillary diameter, *γ*_LG_ is the surface energy at the liquid–gas interface, and *θ*_Adv_ is the advancing contact angle of water on a smooth MOF surface. Taking the average pore diameter of the MOF as 0.6 nm,^45^*P*_c_ was estimated to be ∼181 MPa. On the other hand, to a first approximation, the water hammer pressure can be estimated using eqn (2):

2 *P*_h_ = 0.2*ρCv*

where *C* = 1497 m s^−1^ is the sound velocity in water, *ρ* is the density of water, and *v* is the impact velocity.^46^ Taking *v* = 35 m s^−1^, the *P*_h_ comes out as ∼10.5 MPa which is much lower than the estimated capillary pressure. In other words, these amphiphobic coatings can withstand the water jet impact without any impalement damage. The water hammer pressure is also lower than the mechanical strength of the coating (see Table 1). To demonstrate the superior mechanical robustness of the WPU–MOF coatings, water jet tests were also performed on WPU–SiO_2_ coatings (Fig. S11A†). Commercially available fumed silica nanoparticles (nonporous), incorporated into the WPU matrix (WPU–SiO_2_), was used as a control for comparison. These WPU–SiO_2_ coatings failed in jet impact and clearly showed damage at the impact location (Fig. S11B†), confirming the benefits of MOFs in mechanical integrity. Unlike WPU–SiO_2_, in the case of WPU–MOF coatings, intercalation of polyurethane chains into MOF pores facilitates a more uniform distribution and interlocking at the molecular level which will be expected to help in resisting localised stress concentrations.^47,48^ To verify the intercalation of WPU molecular chains into MOF pores, the dispersion of WPU and MOF nanoparticles was washed several times to remove unbound/loosely adhered WPU, dried (80 °C for 6 h), and subjected to FTIR together with the controls *i.e.* MOF and WPU. As evident from the spectra (Fig. 5F), in addition to the characteristics peaks of the MOF and WPU, a clear shift of the symmetric and asymmetric stretching of –CH_2_ from 2863 cm^−1^ to 2853 cm^−1^ and 2938 cm^−1^ to 2922 cm^−1^ was observed in the spectra of the washed WPU–MOF sample. Further, the broad band centred at 3342 cm^−1^ arises due to the stretching of aliphatic amine groups N–H of polyurethane. This shows the possible van der Waals interaction of the WPU phenyl ring to the MOF structure. Furthermore, solid-state NMR was employed to investigate the interaction between the MOF and WPU. The ^13^C and ^1^H MAS NMR spectra of the WPU, MOF, and WPU–MOF coating are shown in Fig. 5G and H, respectively. The ^13^C MAS NMR spectrum of the MOF contains three characteristic peaks located at isotropic chemical shifts of 129.8, 137.7, and 170.5 ppm. The peak at a high chemical shift is characteristic of C atoms from the carboxylic group. The aromatic C–H and C–C groups in the MOF exhibit a negligible chemical shift (<1 ppm) but a significantly increased line width in the presence of WPU, indicating a strong interaction between the WPU and MOF. Further, the ^1^H NMR spectrum of the MOF contains two main regions (Fig. 5H) characteristic of the aromatic protons (∼8 ppm) and of the Zr–OH groups (0–3 ppm). In the presence of WPU, the line width of the aromatic proton peak increases, which is attributed to the ^1^H–^1^H dipolar coupling of these protons to WPU within sub-nm distances. This indicates the presence of WPU within the MOF pores.^49,50^ These initial signatures of the intercalated WPU–MOF nanocomposite indicate improved interfacial adhesion, reduced particle agglomeration, and enhanced structural reinforcement, leading to increased strength, durability, and impact resistance.

A standard tape peel test (using 3 M VHBTM tape 5952) was also performed to assess the mechanical durability of the coating.^51^ As shown in Fig. 5I, with increasing tape peel cycles, *θ*_Adv_ remains almost the same with a slight increase in Δ*θ*, from 9 ± 2° to 14 ± 2°. No delamination was observed (inset: Fig. 5I), and the coating maintained its repellence even after 50 repetitive tape peeling cycles – again confirming the mechanical robustness of the WPU–MOF coating. However, when subjected to Taber abrasion tests, these coatings exhibited limited wear resistance. This is possibly due to the inherent flexibility of the waterborne polyurethane matrix, which, while beneficial for impact resistance and tensile properties, reduces the coating's ability to withstand continuous abrasive forces. To address this, future work will focus on optimising the formulation, such as by increasing cross-link density or incorporating additional wear-resistant additives, to enhance abrasion resistance while maintaining the desirable functionality of the coating. Additionally, these coatings can retain their structural integrity in acidic environments (pH ∼ 1–2) for 24 hours and under moderately alkaline conditions (pH ∼ 11–12) for up to 12 hours (Fig. S12†).

### 3.5. Thermal stability and ice adhesion measurement

**Fig. 6:**
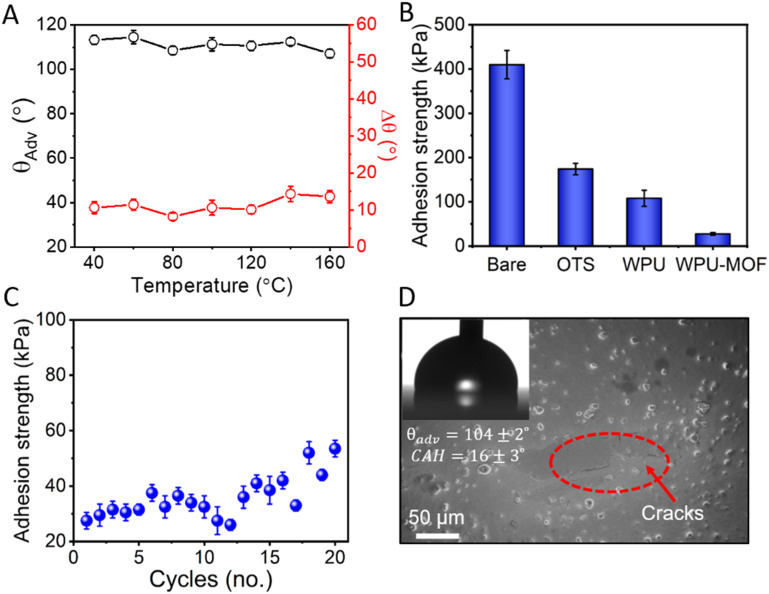
Thermal stability and anti-icing performance. (A) Thermal stability of the coated surface up to 200 °C. (B) Ice adhesion strength of different surface treatments including bare glass as a control, trichlorooctadecyl silane coated glass (OTS), trichlorooctadecyl silane coated WPU on glass (WPU) and our intercalated WPU–MOF coating on glass (WPU–MOF). (C) Variation of ice adhesion strength of the WPU–MOF coating up to 20 icing/deicing cycles. (D) SEM image of the coating showing some cracks after 20 icing/deicing cycles.

The thermal stability of amphiphobic coatings is essential to ensure the adequate performance in outdoor applications such as energy generation and storage (wind turbine blades), chemical and thermochemical processing operations, aviation and power transmission.^52^ The thermal stability of the WPU–MOF coatings was tested in the temperature range of 40 °C to 200 °C; a 2 h exposure was used at each temperature and the change in Δ*θ* was recorded to assess the damage following the cool off of the coating down to room temperature. At the highest tested temperature of 200 °C, Δ*θ* increased slightly from 9 ± 2° to 15 ± 2° and no significant change was observed in the *θ*_Adv_ (Fig. 6A). Therefore, the choice of the porous MOF with very good thermochemical and mechanical robustness contributes favourably. Owing to the smoothness and excellent liquid repellence of the WPU–MOF coating, its anti-icing properties were assessed next by measuring the ice adhesion strength. The WPU–MOF coating was compared with different control surfaces including bare glass, silane coated glass, and silane coated WPU on glass for its ice adhesion strength. As shown in Fig. 6B, the ice adhesion strength of *τ*_ice_ = 30 ± 6 kPa was recorded on WPU–MOF coatings which is ∼92% lower compared to bare glass and well below the threshold to designate it as an ‘icephobic’ surface (*τ*_ice_ < 100 kPa).^53^ For these amphiphobic coatings, the mobility of flexible alkyl silanes might have facilitated the easy removal of ice *via* interfacial slippage. Newby *et al.*^54^ first verified the phenomenon of interfacial slippage and proved that adhesion strengths of a viscoelastic adhesive on a liquid-like silane monolayer is not controlled by thermodynamic work of adhesion, rather depends on the mobility of the grafting silanes. The dynamic, liquid-like behaviour of the silanes reduces interfacial friction, minimises contact points, and weakens adhesion through the reduced effective contact area and dynamic interactions. This results in lower adhesion strength, allowing ice to be removed with minimal force. Furthermore, the robustness of the coating was tested by repeating icing/deicing cycles, followed by measuring ice adhesion strengths after each cycle (Fig. 6C). No significant change in the ice adhesion strength was observed up to 15 cycles. After 20 cycles, the ice adhesion strength increased gradually to 52 ± 8 kPa and some cracks were observed in the coating (Fig. 6D). The *θ*_Adv_ decreased to 104 ± 2° while Δ*θ* increased to 16 ± 3° (see inset in Fig. 6D) after 20 icing/deicing cycles. No significant deterioration of liquid repellency was observed.

## 4. Conclusion

In summary, we report a simple and scalable water-based formulation for non-fluorinated amphiphobic coatings that exhibit comprehensive robustness and optical transparency. The water-based WPU provides excellent adhesion to the substrates, while the intercalation of WPU into the nanohierarchical MOF pores significantly enhances the mechanical durability of the coating. This intercalation, combined with the intrinsic properties of MOFs, endows the coating with superior amphiphobic characteristics. Application of our coatings including the post-functionalisation step is spray based – carefully developed to make it scalable. Previously, except surface-grown MOFs which involved a tedious L/L approach,^22^ amphiphobicity (to a surface tension as low as 25 mN m^−1^) using fluorine-free chemistry remained unachievable.^55,56^ Post-silanisation, the coating offers excellent liquid repellency with low Δ*θ* and roll-off angles for a wide range of low surface tension liquids from alcohols to ketones. The WPU–MOF coating simultaneously demonstrated very good thermal stability, mechanical durability, liquid impalement resistance (∼35 m s^−1^ jet impact), and anti-icing properties (low ice adhesion strength of ∼30 kPa). This water-based spray formulation, with multifunctionality and robustness, offers ample opportunities for different industrial applications. A limitation of the current work is post-functionalisation which makes our approach two-step, which will need to be addressed in future studies. Overall, the present study offers a new approach to synthesise water-based fluorine-free coatings that are not only environmental friendly, but also features the robustness that is necessary for industrial use.

## Data availability

All the materials are provided in the main manuscript and ESI.† For further details, please contact the corresponding author (m.tiwari@ucl.ac.uk).

## Author contributions

Priya Mandal: data curation, analysis, investigation, methodology, writing – original draft, and review & editing. Vikramjeet Singh: supervision and writing – review & editing. Jianhui Zhang: methodology and writing – review & editing. Manish K. Tiwari: conceptualisation, funding acquisition, supervision, and writing – review & editing.

## Conflicts of interest

MKT is involved in commercialisation of liquid-repellent coatings with support from UCLB, UCL's technology transfer office.

## Supplementary Material

EN-012-D4EN00762J-s001

EN-012-D4EN00762J-s002

EN-012-D4EN00762J-s003

EN-012-D4EN00762J-s004

EN-012-D4EN00762J-s005
