# Supplementary material for: Intercalated MOF nanocomposites: robust, fluorine-free and waterborne amphiphobic coatings
Source: Environ Sci Nano. 2025 Jan 29;12(3):1930–41. doi: 10.1039/d4en00762j (PMC11775646; doi:10.1039/d4en00762j)
Supplement: EN-012-D4EN00762J-s005 [file EN-012-D4EN00762J-s005.pdf]

Supplementary Information (SI) for Environmental Science: Nano.
This journal is © The Royal Society of Chemistry 2025

Intercalated MOF Nanocomposites: Robust, Fluorine-free and Waterborne Amphiphobic Coatings

Priya Mandal[1,2], Vikramjeet Singh[1,3], Jianhui Zhang[1,2], Manish K. Tiwari[1,2,3*]

[1]Nanoengineered Systems Laboratory, UCL Mechanical Engineering, University College London, London WC1E 7JE, U.K.
[2]Wellcome/EPSRC Centre for Interventional and Surgical Sciences, University College London, London W1W 7TS, U.K.
[3]Manufacturing Futures Lab, UCL Mechanical Engineering, University College London, London E20 2AE, U.K.

*Corresponding author. Email: m.tiwari@ucl.ac.uk

CONTENTS

Figure S1. Surface morphology of MOF nanoparticles.

Figure S2. PXRD spectra of MOF.

Figure S3. Raman spectra of MOF.

Figure S4. Liquid repellency of WPU-MOF coating at different filler concentrations.

Figure S5. WPU-MOF coating application process on different substrates.

Figure S6. Optimization of transparency of WPU-MOF coating.

Figure S7. SEM image of nanohierarchical MOF embedded in WPU matrix.

Figure S8. Schematic and details of jet impact set-up.

Figure S9. 3D-micropscope image of WPU-MOF coating after repeated jet impacts.

Figure S10. Snapshots of jet impact and 3D-micropscope image of WPU-SiO_2 coating after repeated jet impact test.

Supplementary videos 1-4.

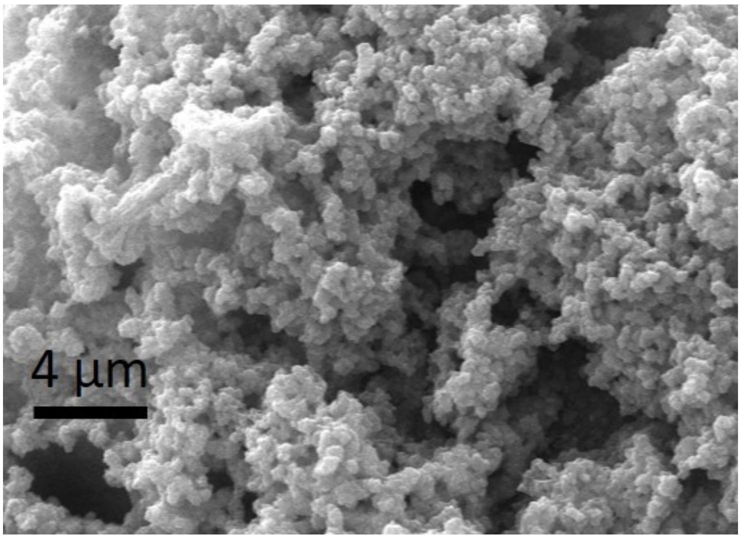

Figure S1. SEM image of as synthesized MOF. The average crystal size is ~100-200 nm.

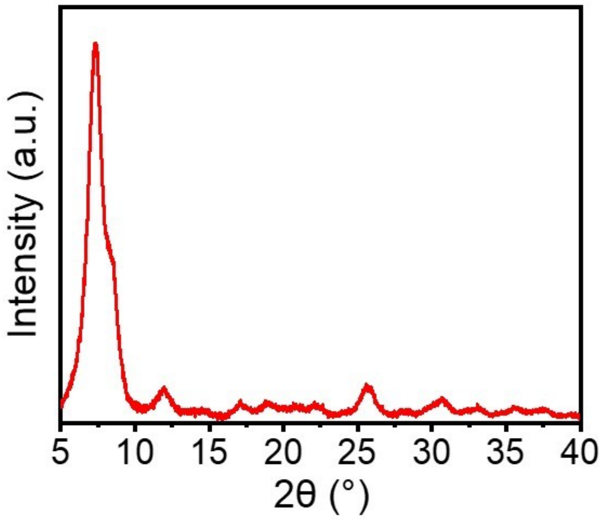

Figure S2. PXRD pattern of MOF.

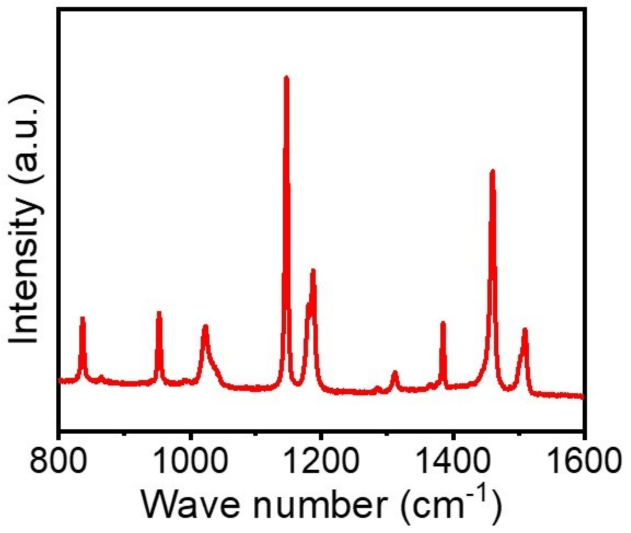

Figure S3. Raman spectra of MOF.

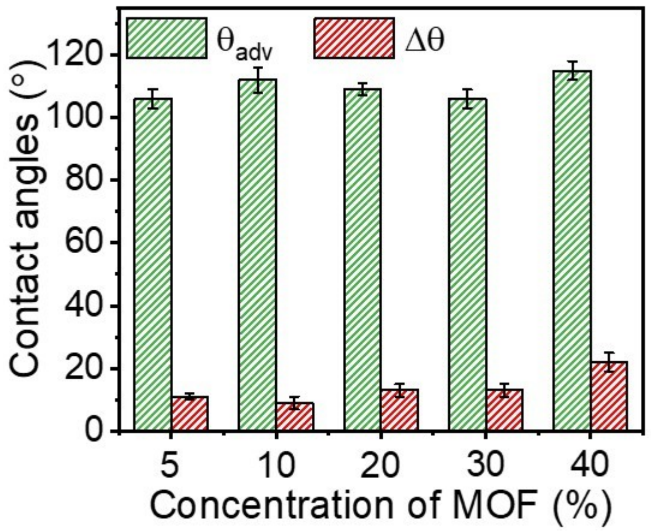

Figure S4. Effect of filler concentration on wettability of the WPU-MOF coating. Error bars represent standard deviation from at least four different measurements at different locations of the coating. WPU-MOF nanocomposites with varying filler concentrations were prepared to determine optimal nanoparticle concentration with best possible liquid repellency.

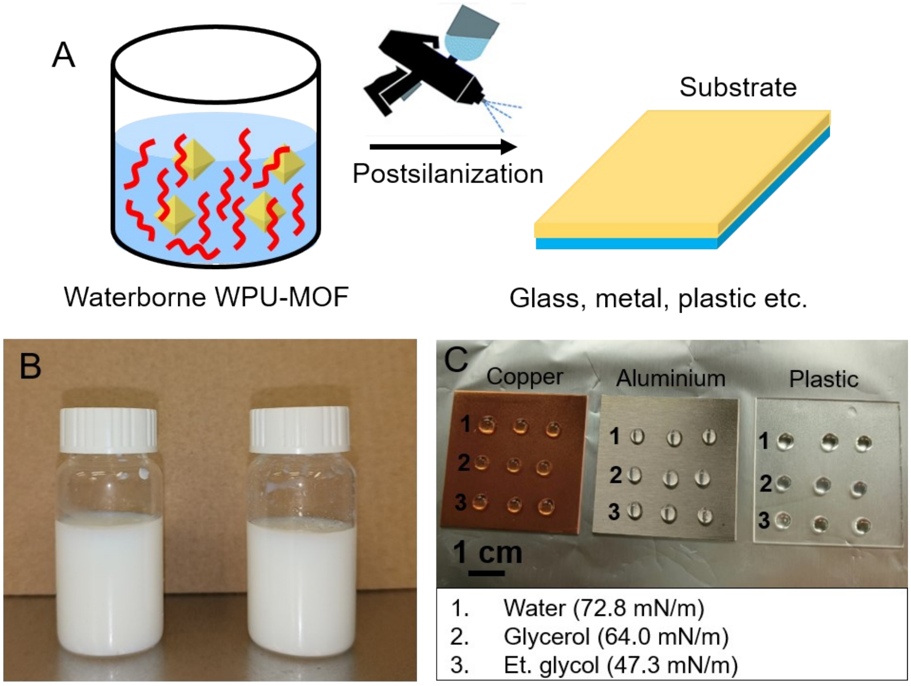

Figure S5. (A) Schematic illustration of WPU-MOF coating via spraying. (B) WPU/MOF/water suspension as prepared (left vial) and stored at room temperature for 1 month (right vial). (C) Optical image of WPU-MOF coating on different substrates: copper (50 mm × 50 mm), aluminium (50 mm × 50 mm), and plastic (50 mm × 50 mm). Liquid droplets of different surface tensions are placed on coated surfaces; 1) Water, 2) Glycerol, and 3) Ethylene glycol.

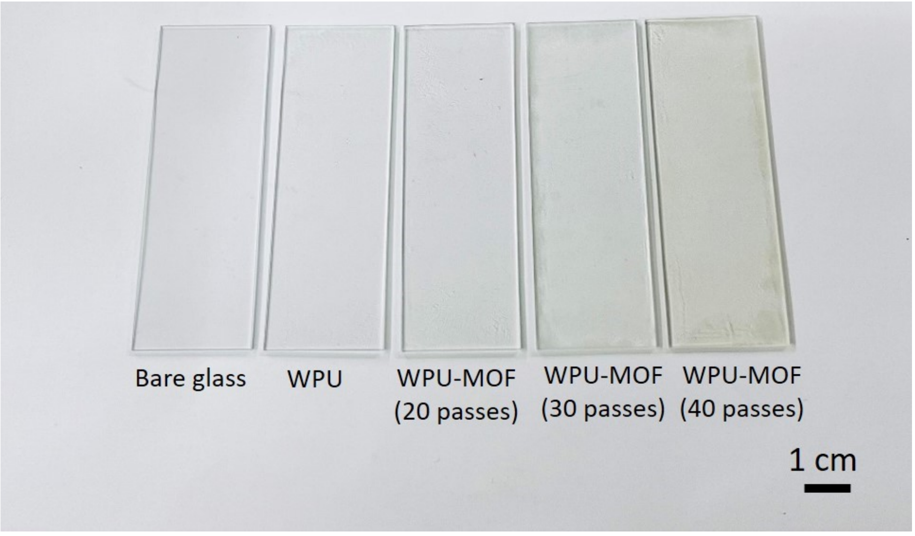

Figure S6. Samples showing effect of spray passes on the transparency.

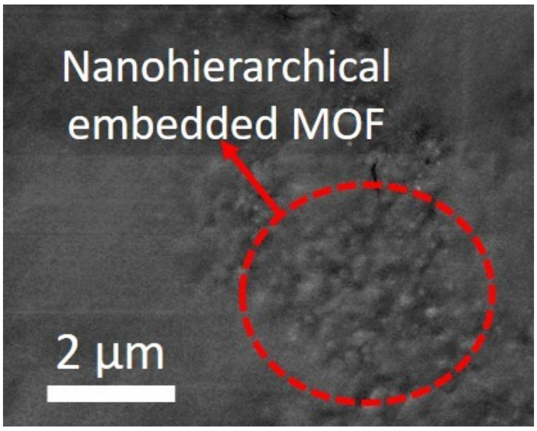

Figure S7. SEM image of nanohierarchical MOF embedded into WPU matrix.

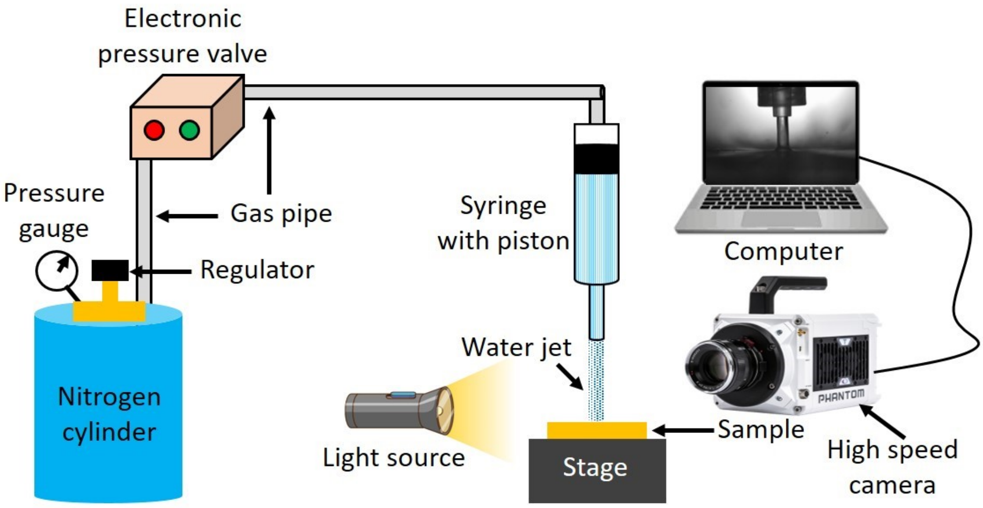

Figure S8. Schematic of water jet impact setup.

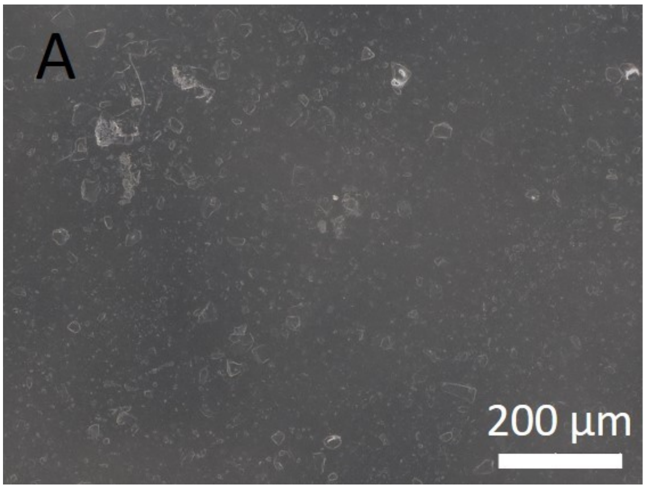

Figure S9. 3D-microscope image of WPU-MOF on glass after repeated jet impacts (3 times) at 35 m/s.

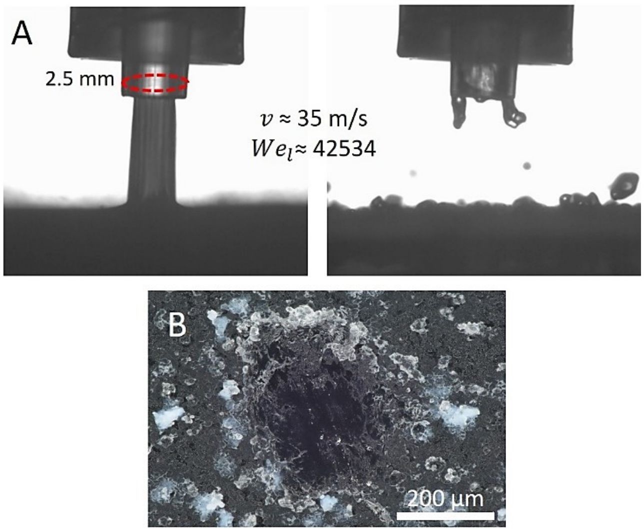

Figure S10. (A) Snapshots of 2.5 mm water jet impacting on WPU-SiO_2 coating vertically with a speed of 35 m/s. (B) 3D-microscope image of WPU-SiO_2 coatings on glass after repeated jet impacts (3 times) at 35 m/s.

SUPPLEMENTARY VIDEO CAPTIONS

Supplementary video 1: Free sliding of water (72.8 mN/m) and low surface tension liquid droplets of glycerol (64.0 mN/m), ethylene glycol (47.3 mN/m), and butanol (25.0 mN/m) on WPU-MOF coated glass at 30° tilt angle.

Supplementary video 2: Sliding of water (72.8 mN/m) and butanol (25.0 mN/m) on silanized WPU coating (without MOF nanoparticles) at 30° tilt angle. Traces can be observed on the surface showing poor repellence with butanol.

Supplementary video 3: A water jet with nozzle diameter 2.5 mm impacted on WPU-MOF coating at different velocity (6 m/s, 18 m/s and 35 m/s) recorded by a high-speed camera. The jet impact test was repeated 3 times at the same spot.

Supplementary video 4: Droplet sliding after repeated (3 times) jet impact test. Free sliding of water droplets confirmed lack of pinning and impalement of the coating.
